# Immune checkpoint inhibitors in the treatment of hepatocellular carcinoma

**DOI:** 10.3389/fimmu.2024.1379622

**Published:** 2024-04-04

**Authors:** Zeynep Akbulut, Başak Aru, Furkan Aydın, Gülderen Yanıkkaya Demirel

**Affiliations:** ^1^ Cancer and Stem Cell Research Center, Maltepe University, Istanbul, Türkiye; ^2^ Department of Medical Biology and Genetics, Faculty of Medicine, Maltepe University, Istanbul, Türkiye; ^3^ Department of Immunology, Faculty of Medicine, Yeditepe University, Istanbul, Türkiye

**Keywords:** hepatocellular carcinoma, immune checkpoint proteins, immune checkpoint inhibition, tumor microenvironment, anticancer immunity

## Abstract

Despite advances in cancer treatment, hepatocellular carcinoma (HCC), the most common form of liver cancer, remains a major public health problem worldwide. The immune microenvironment plays a critical role in regulating tumor progression and resistance to therapy, and in HCC, the tumor microenvironment (TME) is characterized by an abundance of immunosuppressive cells and signals that facilitate immune evasion and metastasis. Recently, anti-cancer immunotherapies, therapeutic interventions designed to modulate the immune system to recognize and eliminate cancer, have become an important cornerstone of cancer therapy. Immunotherapy has demonstrated the ability to improve survival and provide durable cancer control in certain groups of HCC patients, while reducing adverse side effects. These findings represent a significant step toward improving cancer treatment outcomes. As demonstrated in clinical trials, the administration of immune checkpoint inhibitors (ICIs), particularly in combination with anti-angiogenic agents and tyrosine kinase inhibitors, has prolonged survival in a subset of patients with HCC, providing an alternative for patients who progress on first-line therapy. In this review, we aimed to provide an overview of HCC and the role of the immune system in its development, and to summarize the findings of clinical trials involving ICIs, either as monotherapies or in combination with other agents in the treatment of the disease. Challenges and considerations regarding the administration of ICIs in the treatment of HCC are also outlined.

## 1 Introduction

In 2020, liver cancer emerged as a global health problem with 905,700 new cases, accounting for nearly 5% of all cancer diagnoses, and 830,200 deaths, consolidating its position as the third leading cause of cancer-related deaths worldwide, after lung and colorectal cancer. The mortality/incidence ratio, an indicator of the severity of the disease, was reported as 0.92, underscoring the significant burden and poor prognosis associated with liver cancer. In particular, hepatocellular carcinoma (HCC), the predominant form of primary liver cancer, accounted for 75% to 85% of all cases within this category. In the United States, the incidence of HCC has tripled since the 1980s, despite efforts to screen individuals with cirrhosis. Projections indicate a concerning increase in this malignancy worldwide, with an expected 55% increase in new cases between 2020 and 2040, resulting in 1.4 million diagnoses by 2040. There is consensus that HCC will remain a significant and challenging global public health problem for years to come (1).

HCC exhibits a notable gender disparity, affecting men at a rate two to three times higher than women, resulting in higher incidence and mortality rates globally. A compelling risk factor for the development of this malignancy is the presence of cirrhosis due to various liver diseases, a condition observed in over 80% of HCC patients (2). Other documented etiologies include metabolic abnormalities such as α1-antitrypsin deficiency, hemochromatosis, and autoimmune disorders (1).

While cirrhosis stemming from diverse etiologies can promote HCC development, chronic viral hepatitis predominates as the causative factor in over 80% of cases on the global scale (3). Hepatitis B virus (HBV) and hepatitis C virus (HCV) infections remain the main etiological factors in many regions, although their prevalence is decreasing in areas implementing specific programs for the elimination of viral hepatitis (4). At the same time, HCC associated with alcohol abuse and non-alcoholic fatty liver disease (NAFLD) has seen an alarming increase in both incidence and mortality, underlining the requirement for public policies targeting these emerging risk factors to facilitate a sustained reduction in HCC incidence (5). Of particular interest, NAFLD has emerged as the leading cause of HCC even in the absence of cirrhosis, with approximately one third of cases occurring in non-cirrhotic individuals. However, further research is needed to delineate which noncirrhotic NAFLD patients warrant HCC surveillance due to sufficient risk. On the other hand, alcohol-associated cirrhosis stands out as a recognized risk factor for HCC, and the combination of alcohol use with other etiologies increases the risk up to five-fold (6). NAFLD, now a significant public health concern, has become the fastest-growing cause of HCC among liver transplant candidates, closely linked to the escalating prevalence of obesity and metabolic syndrome (7, 8).

Several lifestyle factors besides alcohol use increase the risk of HCC (6). Smoking, for example, is associated with a 20-86% increased risk of HCC, with the risk returning to almost baseline after three decades of cessation (9). Obesity is associated with a 1.5-4.5 times higher risk of HCC and contributes to nearly 10% of HCC cases worldwide (10–12). Components of the metabolic syndrome, particularly diabetes, almost double the risk of HCC in the absence of excess weight (13). Physical activity has also been suggested to have beneficial effects in primary HCC prevention and after cancer diagnosis, over and above the confounding effect of weight loss. In addition, dietary exposure to aflatoxin B1 and aristolochic acid serve as recognized cofactors for HCC in patients with HBV infection.

Currently, therapeutic options for HCC include curative resection, liver transplantation, transarterial chemoembolization (TACE), radioembolization, radiofrequency ablation and chemotherapy, but their efficacy is limited, and they benefit only a small subset of patients (14). Among the approaches abovementioned, surgical resection and liver transplantation are considered as the most effective interventions, although their applicability in the treatment of liver disease is limited. For instance, only 5% of HCC patients are suitable for transplantation (15). Thus, other treatment options may be considered including RFA and TACE. TACE is performed by an interventional radiologist who selectively cannulates the artery feeding the tumor and administers high doses of local chemotherapeutic agents such as doxorubicin, cisplatin, or mitomycin C. However, the impact of TACE on clinical outcomes remains controversial, with some studies suggesting no benefit and others reporting a significant improvement in survival (15). On the other hand, RFA holds significant advantages over solo TACE in terms of initial tumor control, though it has comparable OS and recurrence-free survival with TACE in HCC less than 3 cm in size.

In terms of systemic treatment, introduction of the multi tyrosine kinase inhibitor (TKI) sorafenib has revolutionized HCC management (16). In 2018, TKI Lenvatinib was registered as an alternative for sorafenib in the first-line treatment of the disease (17). In the second-line setting, regorafenib and cabozantinib comprise the backbone of the therapy (18). However, these treatments may be ineffective in advanced stages of HCC and may even lead to progression of the underlying liver disease. Despite encouraging results in preclinical and early clinical trials for certain drugs, there remains a significant gap in effective systemic therapies for advanced stages of HCC. This underscores the urgent clinical need for more robust and targeted interventions to address the challenges posed by advanced liver cancer (14).

## 2 Tumor microenvironment in hepatocellular carcinoma

Immune tolerance in the liver aims to prevent exaggerated responses to harmful stimuli. On the other hand, these tolerance mechanisms also may promote the development and progression of cancer by suppressing immune surveillance. Approximately 80% of HCC cases are associated with persistent inflammation caused by the infiltration of immune cells along with resident cells such as Kupffer cells, hepatic satellite cells (HSCs) and hepatic sinusoidal cells. Prolonged inflammation leads to oxidative stress, creating a microenvironment that induces DNA damage and genetic modifications, paving the way for the initiation and progression of tumor growth (19).

**Figure 1:**
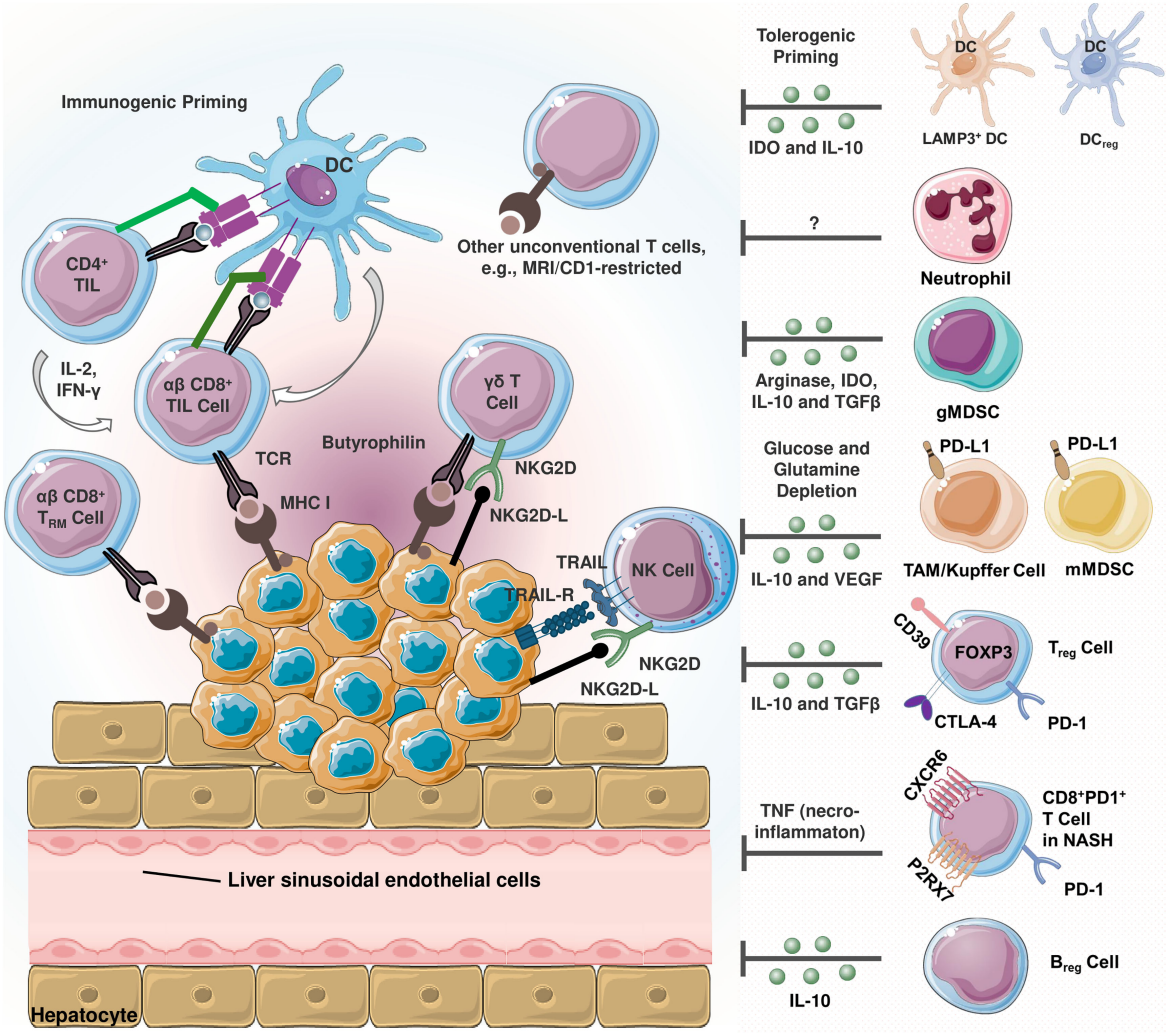
Schematization of infiltrating immune cells in hepatocellular carcinoma (HCC) (20). Immunosuppressive and immunostimulatory cells coexist in the tumor microenvironment (TME). HCC cells express TNF-related apoptosis-inducing ligand (TRAIL) receptor (21). TRAIL promotes natural killer (NK) cell infiltration into the TME, and TRAIL-expressing NK cells exert apoptotic effects on HCC cells. The activating cell surface receptor NKG2D is predominantly found on the surface of cytotoxic immune cells, and its ligands can be expressed in virtually all cell types upon induction including oncogenic transformation (22). Tumor-reactive CD8+ T cells recognize cancer cells via peptide- major histocompatibility complex class I (MHC I) complexes. Once recognized, malignant cells are eliminated via perforin- or FAS-dependent mechanisms. MHC I expression is critical since cancer cells lacking MHC I expression can only be eliminated by NK cells (23). In terms of tolerogenic signaling, regulatory dendritic cells (DCreg) are involved in T cell polarization, myeloid-derived suppressor cells (MDSCs) and T regulatory cell (Treg) differentiation and activity (24). Similarly, lysosome-associated membrane glycoprotein 3 (LAMP3)+ dendritic cells (DCs) are positively correlated with the infiltration of exhausted CD8+ T cells and Tregs (25). In TME, MDSCs are reported to promote tumor progression and are correlated with poor prognosis (26). These cells induce immunosuppression by secreting arginase-1, indoleamine 2,3-dioxygenase (IDO), TGF-β and interleukin-10 (IL-10) (27). Being a significant source of the latter, regulatory cells (Breg) also secrete IL-10 (20). Kupffer cells and other tumor associated macrophages (TAMs) are involved in hepatocarcinogenesis and immune evasion in different mechanisms including secreting immunosuppressive mediators, expressing programmed death-ligand 1 (PD-L1), recruiting Tregs as well as IL-17-expressing CD4+ T helper 17 (Th17), and downregulating MHC II expression along with costimulatory molecules (20). In non-alcoholic steatohepatitis (NASH), a subset of activated CD8+ T expressing exhaustion marker PD-1 are elevated (28). These cells exert an auto-aggressive behavior and drive necro-inflammation by secreting tumor necrosis factor alpha (TNF-α).

In HCC, the tumor microenvironment (TME) is characterized by an increase in immunosuppressive cells including Kupffer cells, M2-type tumor associated macrophages (TAMs), regulatory T cells (Tregs) and myeloid derived suppressor cells (MDSCs) (**Figure 1**) (20–29). Kupffer cells are liver-resident macrophages that are responsible for the phagocytic clearance of pathogens under physiological conditions (30). In case of HCC, these cells can polarize similar to the cancer-promoting TAMs. Kupffer cells and M2-polarized TAMs contribute to immune evasion in HCC through mechanisms such as PD-L1 expression, MHC-II downregulation, secretion of immunosuppressive cytokines, and recruitment of Tregs and CD4+ cells (31). These cells also induce T-cell tolerance by releasing immunosuppressive factors such as interleukin (IL)-10, transforming growth factor (TGF)-β and prostaglandin E2 (PGE-2) (30, 32).

Liver sinusoidal endothelial cells (LSECs) are another resident liver cells, express high levels of PD-L1 and contribute to the induction of Tregs through a TGF-β dependent mechanism (33). These cells are specialized fenestrated endothelial cells that serve as a barrier between parenchymal cells and sinusoidal capillaries, taking part in the removal of blood-borne waste from the systemic circulation and the digestive tract by filtration and endocytosis. Under physiological conditions, fenestrated LSECs contribute to the maintenance of hepatic stem cell quiescence, whereas their capillarized counterparts induce stem cell activation by releasing platelet-derived growth factor (PDGF) and reducing the expression of the protective Kruppel-like factor 2 (KLF2). This process precedes the development of liver fibrosis. Communication between LSECs and other cells in the HCC TME is critical for the progression of liver fibrosis and subsequent HCC development (34).

Characterized as quiescent vitamin A-rich cells, hepatic stellate cells (HSCs) participate in the production of growth factors required for liver development, in addition to amplifying hepatic inflammatory responses (35). In HCC, these cells can acquire a fibrogenic phenotype known as myofibroblastic cells under continuous liver injury and promote fibrosis by altering the ECM (34). They also promote the accumulation of MDSCs and Tregs in the liver, and can induce T-cell apoptosis via PD-1/PD-L1 signaling (36).

In terms of T lymphocytes, these cells are recruited to the tumor site via the chemokine receptor 6 (CCR6) and chemokine ligand 20 (CCL20) axis. A specific subset of MDSCs also induces local differentiation of CD4+ T cells (33, 37). MDSCs contribute to tumor progression through an alternative mechanism involving secretion of vascular endothelial growth factor receptor (VEGFR), which induces vascularization and angiogenesis within the malignant tissue. Other notable players include T helper 17 (Th17) cells, CD4+ T cells expressing CCR4 and CCR6, CD14+ dendritic cells (DCs) expressing CTLA4 and PD1, tumor associated fibroblasts that inhibit NK cell function, and neutrophils that attract macrophages and Tregs (38).

In summary, the TME is composed of various components, including the extracellular matrix, immune cells, helper cells (fibroblasts, HSCs and vascular endothelial cells), cytokines, chemokines and growth factors, which collectively facilitate the immune escape, invasion and metastasis of HCC (39). However, this complexity may also provide potential molecular targets for immunotherapy in the treatment of the disease (39).

## 3 Immune checkpoints in cancer therapy

Neoplastic cells across a broad spectrum of tumor types express immune checkpoint molecules, a phenomenon that has been recognized for its profound impact on the intrinsic biology of these malignancies, particularly regarding their involvement in epithelial-mesenchymal transition (EMT) and related functions. The term “immune checkpoint proteins” (ICPs) refers to the interplay of ligand-receptor pairs that modulate immune responses. In this context, their cognate receptors expressed on immune cells are referred to as “immune checkpoint receptors”, while their counterparts on antigen-presenting cells, tumor cells or other cellular phenotypes are referred to as “immune checkpoint ligands” (40). The vast majority of immune checkpoint molecules characterized to date are expressed predominantly on cells of the adaptive immune system, particularly T cells (**Table 1**). However, it is noteworthy that cells of the innate immune system also contribute to immune checkpoint expression which underscores the complexity and ubiquitous nature of ICPs (77).

**Table 1:** Immune checkpoint proteins, their receptors and/or ligands and their main functions.

ICPs	Cellular Source	Ligands/Receptors	Main Functions	References
PD-1	Activated T cells	PD-L1/PD-L2	1. Blocking the interaction between PD-1 and its ligand. 2. Reducing cytokine secretion.	(15, 41, 42)
PD-L1	DCs, MDSCs, Macrophages	PD-1	Inhibiting T cell responses	(43–45)
CTLA-4	Tregs	CD80/86	Inhibiting T cell responses	(15, 46)
PVRIG	DCs, Th1 cells, NK cells	CD112	Inhibiting T cell responses	(47–50)
TIM-3	DCs, NK cells, Th1 cells, Th17 cells, Macrophages	GAL-9, PS	Inhibiting Th responses	(51–54)
GAL-9	Eosinophils, DCs, T cells, Macrophages, Lymphoid cells, Kupffer cells, intestinal epithelial cells, vascular endothelial cells	TIM-3	Regulating immune homeostasis	(55–57)
VISTA	T cells, APCs	NA	Inhibiting T cell responses	(58–60)
LAG3 (CD223)	Plasmacytoid DCs (pDCs), NK T cells, Tregs	MHC-II, GAL-9, FGL1	lnteracting with MHC-II	(61–63)
TIGIT	T cells, NK cells	CD155, CD112	Supressing anti-tumor immunity	(64–66)
CD40	B cells, DCs, hematopoietic progenitor cells	CD154	Activating NF-κB, MAPKs, PI3, JAK3-STAT5 signaling pathways.	(67–69)
CD70	Activated T cells, mature DCs, B cells	CD27	1. Stimulating T cell differentiation. 2. Enhancing cytotoxic T cell activity. 3. Promoting TNF-α production. 4. Activating B cells.	(70–73)
CD47	RBCs, non-hematopoietic cells	SIRPα, TSP-1, Integrins	1. Activating SHP-1 and SHP-2 pathways. 2. Inhibiting macrophage activity.	(74–76)

SHP1/2, Src-homology 2 domain (SH2)-containing protein tyrosine phosphatase.

ICPs act as gatekeepers that prevent immune system from overreacting, thereby preventing healthy tissue damage and maintaining immune homeostasis during antimicrobial or antiviral immune responses. Unfortunately, the deceptive mimicry of immune checkpoint ligands by cancer cells poses a significant challenge to immune surveillance. The strategic application of immune checkpoint blockade is emerging as a promising approach to attenuate the expression of these ligands on cancer cells, reverse the exhaustion of effector T cells, and restore their potent antitumor functions (78).

ICPs play a pivotal role in inflammatory responses and can be targeted by ICIs for cancer immunotherapy. A group of ICPs, including but not limited to PD-1/PD-L1, CTLA-4, lymphocyte activation 3 (LAG-3), TIM-3, VISTA, and indoleamine 2,3-dioxygenase 1 (IDO1), are shown to be dysregulated in cancer and infectious diseases. These immune checkpoints, along with regulatory cells such as Tregs, MDSCs, M2 macrophages, and cytokines, are upregulated during infection and cancer, effectively altering the immunological milieu. Cancer cells disrupt the immune response and evade immune surveillance by dysregulating immune checkpoint signaling. Blackburn et al. have demonstrated that T-cell function is attenuated with increased expression of immune checkpoints, highlighting the potential of targeted modulation of these ICPs for cancer immunotherapy (79).

ICPs are closely related to and co-evolved with stimulatory immune receptors. These receptors often rely on monotyrosine signaling motifs, specifically the immunoreceptor tyrosine-based inhibitory motif (ITIM) and the immunoreceptor tyrosine-based switch motif (ITSM), to transduce inhibitory signals. As cell-surface molecules, their functional activity is highly susceptible to inhibition by the strategic use of blocking antibodies that interfere with ligand-receptor binding. In the therapeutic field, ICP blockade is emerging as a pioneering approach, demonstrating resilience and longevity that surpasses conventional chemotherapy or targeted therapies. This enhanced durability may reflect the intricate machinery of the immune system’s intrinsic memory. Among the broad spectrum of immune checkpoint blockade therapies, the outstanding success story unfolds with anti-PD-1/PD-L1 therapy, a therapeutic approach that has been approved for the treatment of a diverse array of cancers spanning hematologic, cutaneous, pulmonary, hepatic, vesical, and renal malignancies. The remarkable success of this approach underscores its efficacy in treating a broad spectrum of malignancies (80–82).

### 3.1 Programmed death – 1

PD-1 is expressed on activated T cells and is known to play a key role in immune tolerance. It recognizes two ligands, PD-L1 and PD-L2, which are expressed at low levels in normal tissues but at aberrant levels in certain tumors (15). PD-L1 is ubiquitously expressed on various cells, including B cells, T cells, macrophages, tumor cells and non-immune tissue cells such as vascular endothelial cells (83, 84). The interaction between PD-1 and PD-L1 can induce T-cell dysfunction and anergy, facilitating the escape of PD-L1-expressing tumor cells from cytotoxic T-cell-mediated cell death (41). PD-1 engagement also reduces cytokine secretion, including IL-2, IFN-γ and TNF-α, and inhibits cell proliferation by disrupting the CD28 costimulatory pathway (42). Notably, both tumor and immune cells can express PD-L1, which serves as a valuable biomarker for predicting response to anti-PD-1/PD-L1 axis blockade in various cancers (85). PD-L1, also known as B7-H1 or CD274, contributes to the inhibition of the cancer-immunity cycle by binding to negative regulators of T-cell activation such as PD-1 and B7.1 (CD80) (46).

### 3.2 Programmed death ligand – 1

PD-L1 has a molecular structure similar to other B7 molecules and conforms to the typical architecture of the immunoglobulin superfamily. PD-L1 is classified as a type I transmembrane glycoprotein with an extracellular domain that has an immunoglobulin structure that includes both an Ig variable (V) distal region and an Ig constant (C) proximal region. The hydrophobic transmembrane sequence anchors PD-L1 to the cell membrane, followed by a short intracytoplasmic region with minimal sequence similarity to other B7 molecules. However, this intracellular region contains three conserved sequences - the RMLDVEKC, DTSSK and QFEET motifs - that are shared among mammalian PD-L1 molecules. Furthermore, accumulating evidence suggests that this region plays a pivotal role in survival signaling, with a particular focus on the functions associated with the RMLDVEKC and DTSSK motifs, as demonstrated in recent studies (43). PD-L1 is continuously expressed at varying levels on cells that belong to the myeloid lineage, including DCs, macrophages, and MDSCs. In addition, PD-L1 is found in other cell types beyond the myeloid lineage, including numerous tumors and cancer cell lines. In cancer, pro-inflammatory stimuli such as interferon gamma (IFN-γ) released by T cells have been shown to stimulate PD-L1 expression. This induction is mediated by activation of the Janus kinase (JAK) signal transducer and activator of transcription (STAT) pathway, which ultimately leads to upregulation of interferon regulatory factor 1 (IRF1). IRF1, in turn, binds to the PD-L1 promoter and contributes to the increased expression of PD-L1 (44). Along with the pro-inflammatory cytokine tumor necrosis factor alpha (TNF-α), IFN-γ leads to the activation of the NF-κB pathway, promoting the transcriptional transactivation of PD-L1. This interaction between these pathways not only provides a sophisticated mechanistic understanding, but also sheds light on the elevated expression levels of PD-L1 in inflamed tissues. This phenomenon is particularly observed in extensively infiltrated “hot” tumors (45). PD-L1 transcription also relies on the cell type and the physiological and pathological situation, for example, in HCC, SOX2 in reported to regulate PD-L1 expression (86).

### 3.3 Cytotoxic T-lymphocyte-associated protein 4

Cytotoxic T-lymphocyte-associated protein 4 (CTLA-4), a protein receptor predominantly expressed on T cells, was initially recognized as a secondary receptor for the T-cell costimulatory molecule B7, but subsequently revealed its role as a negative regulator of T-cell activity (46). The mechanism of this regulatory action begins with the immediate upregulation of CTLA-4 upon T cell receptor (TCR) engagement, reaching a higher point 2 to 3 days after activation (87). CTLA-4 has two ligands, CD80 and CD86, also known as B7-1 and B7-2 which shares similarities with T cell costimulatory protein CD28. Both CD28 and CTLA-4 bind to as B7-1 and B7-2, and their binding kinetics coupled with differential avidities result in rapid competitive inhibition by CTLA-4. In addition, CTLA-4, encased in intracellular vesicles, makes a rapid journey to the immunological synapse upon T cell activation. Upon interaction, B7 ligand binding stabilizes CTLA-4, allowing it to accumulate and effectively outcompete CD28 (15).

The story of CTLA-4 continues to unravel with the revelation that its inhibition not only enhances a spectrum of helper T cell-dependent immunological responses, but also interacts in a complex manner with Tregs to amplify their suppressive capacity. Constitutively produced by Treg, CTLA-4, a target gene of the forkhead transcription factor FOXP3, orchestrates the Treg responses, though the exact mechanism remains unclear (88). Interestingly, how CTLA-4 drives the immunosuppressive activity of Treg cells remains a mystery. Thus, the dual aspects of enhanced effector CD4+ T cell activity and attenuation of Treg cell-dependent immunosuppression are key elements in the mechanism of CTLA-4 blockade (80).

### 3.4 Poliovirus receptor-related immunoglobulin domain-containing

Poliovirus Receptor-Related Immunoglobulin Domain-Containing (PVRIG, also called as CD112R), a poliovirus receptor-like protein and has been recognized as a novel co-inhibitory receptor for human T cells as well as NK cells, with a higher affinity for interaction with CD112 compared to CD226 and TIGIT (47). PVRIG has also been shown to be expressed in certain types of cancer, and the highest expression levels in terms of cancer tissues have been reported in kidney, ovary, lung, prostate, and endometrium (48, 89, 90). Moreover, in a study published by Zhu et al., the authors have revealed that it is also expressed on DCs, playing a pivotal role in mediating interactions with DCs and tumor cells through its engagement with PVRIG (90). Disruption of this interaction has been shown to enhance T cell functions, as in TILs, PVRIG expression along with PD-1 and TIGIT has been reported on CD8+ and CD4+ T cells, in correlation with an exhausted phenotype (48). Similarly, PVRIG expression together with CD96, TIGIT, Tim-3 and PD-1 was observed in NK cells (49, 50).

### 3.5 T cell immunoglobulin and mucin domain-containing protein 3

T cell immunoglobulin and mucin domain-containing protein 3 (TIM-3) is a versatile immune checkpoint receptor that plays a central role in the regulation of immune responses. Being a member of the TIM family, TIM-3 is expressed on various immune cells, including IFN-γ-producing Th1 CD4+ and CD8+ T cells, Th17 cells, Tregs, NK cells, DCs, and macrophages (51–53).

TIM-3 binds to several ligands, in particular galectin-9 (Gal9) and cell surface phosphatidylserine (PS) (54). The interaction between TIM-3 and Gal9 or high-mobility group protein B1 (HMGB1) initiates an inhibitory signal that induces apoptosis of Th1 cells. Notably, prolonged exposure to interleukin-12 induces TIM-3 expression on T cells in the tumor microenvironment, leading to functional impairment and exhaustion. In addition to its role on T cells, TIM-3 on immune cells such as natural killer cells and DCs plays a critical role in immune regulation. For example, TIM-3 regulates the differentiation and immunogenic activities of natural killer cells. In addition, when expressed on DCs, TIM-3 facilitates the phagocytosis of apoptotic cells through PS interaction, thereby enhancing antigen presentation and inducing immune tolerance. At the same time, TIM-3 negatively modulates the innate immune system through pattern recognition. Interestingly, TIM-3 cooperates with Toll-like receptors to induce inflammation by activating the transcription factor nuclear factor kappa B and increasing the secretion of pro-inflammatory mediators, revealing its multifaceted role in immune modulation (54).

### 3.6 V-domain immunoglobulin suppressor of T cell activation

VISTA, a type I transmembrane protein, has a structural composition comprising a single N-terminal immunoglobulin (Ig) V domain, a connecting stalk of approximately 30 amino acids, a transmembrane domain, and a cytoplasmic tail of 95 amino acids (91). Studies regarding VISTA’s IgV domain reveal remarkable homology with PD-L1, highlighting a shared structural similarity. Interestingly, the conserved cytoplasmic tail of VISTA mirrors the features of CD28 and CTLA-4 but lacks the conventional ITIM/ITAM motifs commonly found in other B7 co-receptor molecules. Despite the absence of the abovementioned motifs in its cytoplasmic domain, VISTA exhibits potentially functional elements such as protein kinase C binding sites and a proline-rich motif. These structural features suggest that VISTA may serve as a platform for interaction with various protein complexes. The idea that VISTA acts as a ligand is supported by experimental observations, in particular the inhibitory effects of a VISTA-Ig fusion protein on the proliferation of mouse and human CD4 and CD8 T cells, as well as the production of key cytokines such as IFN-γ and IL-2 upon anti-CD-3 stimulation (58, 59). This dual role underscores VISTA’s ability to function as both a ligand and a receptor, underlining its importance in the regulation of immune responses (59).

VISTA has been traditionally recognized for its role in suppressing T cell-associated responses, contributing to immune escape and survival in several human cancers, including prostate cancer, non-small cell lung cancer (NSCLC), colorectal cancer (CRC), acute myeloid leukemia (AML), pancreatic cancer, cutaneous melanoma, metastatic melanoma, hepatocellular carcinoma, ovarian cancer, oral squamous cell carcinoma, and gastric cancer. However, the complex effects of VISTA on cancer immunity go beyond the initially perceived suppressive role. Compelling evidence challenges the simple classification of VISTA as an immunotherapy target, showing that in certain cancers, VISTA assumes stimulatory checkpoint-like functions and actively participates in the activation of anti-cancer immune responses. This complexity underscores the nuanced and controversial nature of VISTA’s role in immune regulation (60).

### 3.7 Lymphocyte activation gene 3

LAG3, also known as CD223, was discovered in 1990 and is a transmembrane molecule expressed on various immune cell types, including CD4+ and CD8+ T cells, NK T cells, NK cells, plasmacytoid DCs (pDCs), and Tregs (61, 62). It is noteworthy that pDCs and Tregs exhibit continuous expression of LAG3, while in other cell types, LAG3 expression is typically induced upon activation (62). Located on human chromosome 12 (12p13), the LAG3 gene shares a genomic region with the CD4 gene, although their protein-level homology is less than 20% (61, 92). LAG3 protein has a molecular weight of 70 kDa, interacts with major histocompatibility complex II (MHC-II) on antigen-presenting cells (APCs) with a significantly higher affinity than CD4 (61, 63).

The structural composition of LAG3 includes an extracellular region, a transmembrane region, and an intracellular region. The extracellular portion consists of four immunoglobulin superfamily domains, specifically a V region and three C2 regions. The V region is distinct, with an extra ring in the middle and an abnormal in-chain disulfide bridge. Meanwhile, the cytoplasmic region of LAG3 consists of three elements: a serine phosphorylation site, a conserved ‘KIEELE’ motif, and a glutamate-proline-dipeptide repeat (EP) sequence. The ‘KIEELE’ motif is highly conserved and exclusive to LAG3, and it takes part in LAG3 related inhibitory signaling (93). In summary, LAG3 is a multifaceted immune regulator with a unique structural profile that expresses dynamic interactions with MHC-II and contributes to intracellular signaling through its distinctive cytoplasmic motifs (93).

### 3.8 T cell immunoreceptor with Ig and ITIM domains

TIGIT, also known as WUCAM, VSTM3 and VSIG9, is identified in 2009 as a co-inhibitory receptor belonging to the immunoglobulin superfamily which consists of an extracellular domain harboring an immunoglobulin variable region (IgV) linked to a type 1 transmembrane domain, and an intracellular domain containing an immunoreceptor tyrosine-based inhibitory motif (ITIM) and an Ig tail-tyrosine (ITT)-like motif constitute (94). Activated CD4+ and effector CD8+ T cells and NK cells express TIGIT on the cell surface, which interacts with the poliovirus receptor (PVR, also known as CD155) with high affinity and with poliovirus receptor-related 2 (PVRL2, also known as CD112) with lower affinity (64). TIGIT shares these ligands with two other receptors, CD226 (DNAM-1) and CD96 (TACTILE), which transmit co-stimulatory and co-inhibitory signals, respectively (64).

TIGIT expression in humans is a late event in the cancer-immunity cycle, occurring after chronic exposure to tumor antigens (79, 95). TIGIT is found on various immune cells that infiltrate tumors in diseases such as melanoma, NSCLC, CRC, HCC, gastric cancer, glioblastoma and hematologic malignancies. In cases such as follicular lymphoma, increased numbers of TIGIT-expressing CD4+ and CD8+ T cells within tumors are reported to be correlated with worse outcome (65). In AML, high TIGIT expression on peripheral blood CD8+ T cells is associated with treatment resistance (66). In addition, the presence of PD-1+TIGIT+CD8+ T cell populations in the blood is negatively correlated with overall survival and progression-free survival in patients with hepatitis B virus-associated HCC (HBV-HCC) (96). Altogether, these findings suggest that TIGIT plays a suppressive role in anti-tumor immunity in cancer patients.

### 3.9 Galectin-9

Galectins are a family of β-galactoside-binding proteins that are not only found in animals, but also in bacteria and fungi to varying degrees. Characterized by an evolutionarily conserved carbohydrate recognition domain (CRD), these proteins share a highly conserved core sequence. Initially recognized for their role in identifying endogenous (“self”) carbohydrate ligands during embryogenesis and early development, galectins have since been found to play critical roles in tissue repair, adipogenesis, cancer development, and regulation of immune homeostasis. The galectin protein family shares two characteristics: a conserved amino acid sequence with significant similarities and a strong affinity for β-galactoside sugars. To date, 15 galectins have been identified in mammals, 11 of which are expressed in humans (55).

Unlike other members of the galectin family, Galectin-9 (Gal-9) acts as an inhibitor of the immune system. Its function includes promoting the differentiation of Tregs while decreasing Th17 and Th1 cells. This dual action contributes to the suppression of excessive immune responses and inflammation (56, 97). Gal-9 selectively engages its receptor, TIM-3, leading to apoptosis in CD8+ T cells. In addition, this interaction initiates adaptive immune responses by promoting the secretion of IL-12 (56, 57).

In CRC, Gal-9 expression was found to be lower compared to para-cancerous tissues, and a positive correlation between low levels of Gal-9 expression and poor prognosis, including lower histologic grade and the presence of lymph node metastasis, was reported (55, 98). In breast cancer, Gal-9 has been shown to have anti-metastatic potential, most likely by inducing tumor cell aggregation and reduced adhesion of breast cancer cells to the extracellular matrix, thus preventing metastasis and improving patient survival (55, 99).

### 3.10 CD40

Identified four decades ago, CD40 is a membrane protein found on B lymphocytes, DCs, hematopoietic progenitor cells, epithelial cells, and tumor cells. This 45-50 kDa glycoprotein consists of 277 amino acids and is a member of the tumor necrosis factor receptor (TNFR) superfamily (67, 100). The ligand of CD40 is CD40L (CD154), a 32-39 kDa type II transmembrane protein that belongs to the TNF superfamily and has a distinct extracellular structure with a β-sheet, α-helix loop and another β-sheet. This structure allows CD40L to form trimers, a feature shared with other ligands in the TNF family. CD40L is primarily expressed by activated T cells, B cells and platelets, but is also expressed by monocytes, NK cells, mast cells and basophils under inflammatory conditions. There is also a soluble form of CD40L that participates in similar actions with its membrane-bound counterpart. CD40 signaling relies primarily on adaptor proteins known as TNF receptor-associated factors, which subsequently activate both the canonical and noncanonical NFκB pathways, as well as the MAP kinase, PI3 kinase, and phospholipase C-γ pathways. When activated, these pathways result in diverse downstream effects, including activation of gene transcription, reorganization of the cytoskeleton, and promotion of cell survival. It has also been reported that CD40 can transmit signals through the JAK3-STAT5 pathway, and when this signaling is absent, DCs promote T cell tolerance. However, further studies are still required to unveil the precise contributions of these pathways, either individually or in combination, to the diverse functional activities of DCs and their differentiation (68).

CD40 binding on the surface of DCs has been shown to promote their cytokine production, induce costimulatory molecules on their surface, and facilitate antigen cross-presentation, eventually “licensing” them to mature, and effectively initiate T cell activation and differentiation. In B cells, CD40 signaling promotes germinal center (GC) formation, immunoglobulin (Ig) isotype switching, somatic hypermutation (SHM) of Ig to increase antigen affinity, and ultimately the generation of long-lived plasma cells and memory B cells. In addition, the CD40 signaling pathway is critical for the survival of several cell types, including GC B cells, DCs, and endothelial cells, both under normal conditions and during inflammation. Dysregulation of CD40 signaling has been observed in autoimmune diseases (69).

In terms of cancer, CD40 expression is observed in 80% of NSCLC cases, 40% of ovarian cancer cases, and 68% of pancreatic adenocarcinoma cases in a recently published study (101). However, it was not found to be prognostic for overall survival for these cancers. On the contrary, cytoplasmic CD40 expression was reported to be positively correlated with higher overall survival, although there was a higher ratio of positive cases in cancer cases in comparison with the normal tissue (102).

### 3.11 CD70

CD70 is a member of the tumor necrosis factor (TNF) family which is exclusively expressed on activated T cells, B cells, and mature DCs (70). It plays a critical role in the immune response by interacting with its receptor CD27, which is expressed on naive T-cells, memory B-cells, NK-cells, and hematopoietic stem and progenitor cells (71, 72, 103). Being a transmembrane phosphoglycoprotein, CD27 functions as a co-stimulatory immune checkpoint receptor that is consistently present on various T cells (including naive, αβ, γδ, and memory T cells), NK cells, and B cells. Upon CD70 binding, CD27 engages TNF receptor-associated factors (TRAFs) and initiates intracellular signaling that enhances the survival and activation of T, B, and NK cells through TRAF2 and TRAF5 signaling, in addition to activating the NF-κB pathway. The CD70-CD27 pathway not only actively stimulates T cell expansion and differentiation, but also enhances CD8+ T cell cytotoxic activity and promotes T cell TNF-α production (70, 73). In addition, CD27-CD70 signaling has also been shown to induce B-cell activation, their terminal differentiation to plasma cells and in addition to increasing NK-cell activity via IFN-γ and IL-2 (73).

Under physiological conditions, the interaction between CD27-CD70 is tightly controlled to prevent overexpression and subsequent excessive lymphocyte activation (104). In contrast to its restricted expression in normal tissues, CD70 is aberrantly expressed in cancer: in oncology, CD70 is often overexpressed in malignant cells, either independently (solid tumors) or along with CD27 (hematological malignancies) (73, 105). To date, several studies have highlighted the CD70-CD27 signaling axis as a key driver of malignancy in hematological cancers, controlling the regulation of processes such as stemness, proliferation and survival. In addition, the importance of CD70 in solid tumors has become apparent, with aberrations reported in several types of cancer, including renal cell carcinoma, nasopharyngeal carcinoma, glioblastoma, melanoma, lung carcinoma, cervical carcinoma, breast carcinoma, ovarian carcinoma and mesothelioma, all of which are associated with decreased survival (70, 106–109).

### 3.12 CD47

Identified as a transmembrane protein present on red blood cells (RBCs), CD47 is a 47-50 kDa membrane protein currently known to be expressed by a variety of healthy cells in addition to cancer cells (74–76). Among the various ligands of CD47; SIRPα, TSP-1, and integrins are the most studied (74).

SIRPα belongs to the signal regulatory protein (SIRP) family and is characterized by an intracellular domain containing an immunoreceptor tyrosine-based inhibitor motif (ITIM), a transmembrane spanning region, and three extracellular immunoglobulin superfamily domains. When CD47 binds to SIRPα, the ITIM in the cytoplasmic tail of SIRPα is phosphorylated. This event recruits and activates phosphatases, including Src homology phosphatase (SHP)-1 and SHP-2. Notably, SIRPα is predominantly expressed on myeloid cells such as monocytes, granulocytes, DCs and especially macrophages. The interaction between CD47 and SIRPα serves as a mechanism to distinguish self from non-self. When this binding occurs, it triggers a “don’t eat me” signal that inhibits macrophages from phagocytosing the adherent cells. In essence, the CD47-SIRPα interaction acts as a regulatory mechanism to prevent macrophages from engulfing healthy cells (74).

Its role in maintaining immune homeostasis makes CD47 an important target for cancer therapy. In the field of oncology, CD47 was first identified as a tumor antigen in human ovarian cancer and has since been found to be overexpressed in several malignancies, including non-Hodgkin’s lymphoma, T-cell lymphoma, AML and myelodysplastic syndrome (MDS). CD47 has the ability to interact with certain extracellular ligands, including SIRPα, thrombospondin-1 (TSP-1), integrins (α2β1, α4β1, α5β1, and α6β1), SIRPγ, CD36, and CD95 (74). The potential of CD47 as an important checkpoint in cancer therapy stems from its critical role in balancing the inhibitory and stimulatory functions of myeloid cells. CD47 engagement induces tumor cell apoptosis through a caspase-independent mechanism. In addition, blocking CD47 leads to phagocytic uptake of tumor cells by antigen-presenting cells, facilitating subsequent antigen presentation to T cells. In addition, anti-CD47 not only neutralizes the inhibitory effect of TSP-1 on human NK cells, but also enhances NK cell activation and cytotoxicity. Early phase clinical trials have shown promising results for CD47 blockade in various cancers, either as a single agent or in combination with other agents. A preclinical study highlighted that the therapeutic effect of CD47 blockade is based on the STING pathway, which induces a type I/II interferon (IFN) response mediated by DCs and CD8+ T cells. Finally, there is evidence in the literature that the CD47/TSP-1 pathway has diverse effects on the immune system and represents a novel target for potential cancer therapeutics (110).

## 4 Targeting immune checkpoints for the treatment of hepatocellular carcinoma

HCC is staged and treated according to the Barcelona Clinic Liver Cancer (BCLC) staging system. This classification divides the disease into four stages: (very) early stage (BCLC stage 0/A), which is the only potentially curable stage; intermediate stage (BCLC stage B); advanced stage (BCLC stage C); and end-stage (BCLC stage D). Unfortunately, approximately 75% of patients are diagnosed at a non-curative stage, limiting treatment options to local interventions (BCLC stage B) and systemic treatments (BCLC stage C). This underscores the importance of tailoring therapeutic approaches based on the specific stage of HCC to optimize patient outcomes (111). Over the past thirteen years, there have been significant advances in the systemic treatment of HCC. The landscape was transformed in 2007 with the introduction of sorafenib, a potent multi-TKI, which maintained its prominence in systemic therapy for over a decade. In 2018, lenvatinib, another TKI with similar properties, was approved as an alternative to sorafenib for the first-line treatment of the disease. During this time, an increasing number of patients with HCC were being treated with lenvatinib. In the second line setting, regorafenib, cabozantinib and ramucirumab have emerged as successful additions to the HCC treatment options, contributing to the evolving landscape of therapeutic strategies for the treatment of this disease (**Table 2**) (111).

**Table 2:** Clinical trials regarding ICI as monotherapies in HCC treatment.

ICIs	Patients (n)	Disease	mOS	ORR(%)	References
Nivolumab	371	Advanced HCC	16.4	15.4	(112)
Pembrolizumab	278	Advanced HCC	13.9	18.2	(113)
	104	Advanced HCC	12.9	12.9	(114)
Tislelizumab	674	Advanced HCC	15.9	15.9	(115)
Toripalimab	36	Advanced HCC	NR	63.9	(116)
Sintilimab	380	Advanced HCC	10	25	(117)
	36	Advanced HCC	15.9	36.1	(118)
Camrelizumab	217	Advanced HCC	6	14.7	(119)
Spartalizumab	74	HCC	NR	NR	(120)
Cemiplimab	26	Unresectable HCC	3.7	19.2	(121)
	21	Resectable HCC	12.4	15	(122)
Atezolizumab	59	Unresectable HCC	6.6	36	(123)
Atezolizumab and Bevacizumab	336	Advanced HCC	12	67.2	(124)
Durvalumab	24	Advanced HCC	NR	83.3	(125)
	47	Unresectable HCC	NR	21.3	(126)
	389	Unresectable HCC	16.6	17	(127)
Avelumab	30	Advanced HCC	4.4	10	(128)
	22	Advanced HCC	14.1	13.6	(129)
	33	Advanced HCC	17.2	55	(130)
Ipilimumab and Nivolumab	49	HCC	12.8	31	(131)
Tremelimumab	21	HCC	8.2	NR	(132)
	32	HCC	12.3	NR	(133)
	39	HCC	10.9	NR	(134)
Tremelimumab and Durvalumab	40	Unresectable HCC	NR	15	(135)
Cobalimab	42	HCC	NR	46	(136)

ICI, Immune Checkpoint Inhibitors; n, Number; mOS, Median Overall Survival; ORR(%), Overall Response Rate; HCC, Hepatocellular Carcinoma; NR, Not reported.

Recent advances in immunotherapy and innovative combinations have reshaped the treatment landscape for HCC while ongoing clinical trials continue to illuminate the way forward. Immunotherapy has demonstrated the ability to improve survival and achieve durable cancer control in certain groups of HCC patients, while mitigating adverse side effects. This represents significant progress in tailoring treatments to improve outcomes in the treatment of this cancer (14).

### 4.1 Nivolumab

Nivolumab is the first anti-PD-1-antibody and demonstrated efficacy as a second-line treatment for patients with HCC in the Phase 1/2, open-label CheckMate040 trial, which enabled its accelerated approval of the drug by the FDA in September 2017. The study enrolled a total of 214 patients, including those with HCV/HBV, in addition to patients who received sorafenib and sorafenib näive (137). In the patient cohort, 20% (42 patients) had an objective response regardless of prior treatment with sorafenib, with three patients achieving a complete response. In addition, 67% (144 patients) had disease that had spread beyond the liver and 29% (63 patients) had major blood vessel involvement. A favorable disease control rate was observed in 64% (138 patients). A total of 48 patients discontinued treatment, with 25% (12 patients) experiencing grade 3/4 treatment-emergent adverse events. These results led to the initiation of the Phase 3 CheckMate459 trial, which was designed to evaluate the efficacy of nivolumab as a first-line treatment to demonstrate superiority over sorafenib (138). A total of 743 patients were enrolled in this study and overall survival was reported to be 16.4 months for nivolumab and 14.7 months for sorafenib. First-line treatment with nivolumab did not show a significant improvement in overall survival compared to sorafenib, although it demonstrated positive clinical activity and a favorable safety profile in patients with advanced HCC. Therefore, nivolumab may be considered as a therapeutic option for patients for whom TKIs and antiangiogenic agents are contraindicated or carry significant risks (112).

### 4.2 Pembrolizumab

Pembrolizumab, a humanized IgG4 monoclonal antibody, is the second anti-PD-1 antibody to be approved in a range of solid tumors and was evaluated extensively for its potential use in the treatment of HCC (113, 114, 139). In a Phase 2 trial, 29 patients were enrolled where they were treated with 200 mg pembrolizumab in three-week cycles. The primary goal of this study was to assess the drug’s efficacy in patients with unresectable HCC. Results of this study revealed that pembrolizumab was effective in the treatment of advanced HCC while its toxicity was generally tolerable and reversible. In addition, analysis of immunological markers in blood plasma, along with PD-L1 staining, suggested that baseline TGF-β levels could serve as a potential predictive biomarker for determining the response to pembrolizumab (139, 140). In KEYNOTE-224, 169 patients were screened and 104 were selected to receive pembrolizumab every three weeks for approximately two years or until disease progression. In KEYNOTE-224, 169 patients were screened and 104 were selected to receive pembrolizumab every three weeks for approximately two years or until disease progression. The overall response rate was reported as 18 out of 104 patients, with one patient achieving a complete response (1%) and 17 patients achieving a partial response (16%) (114). A total of 413 patients were enrolled in KEYNOTE-240 and received pembrolizumab every 3 weeks for approximately 2 years. The primary endpoint of the study was progression-free survival. Although the results of this study were consistent with those of KEYNOTE-224, overall survival and progression-free survival did not reach statistical significance in this study (113). Combinatorial administration of pembrolizumab with levantinib resulted in a remarkable overall response rate of 46% where among all patients with unresectable HCC who had not previously undergone systemic treatment, 11% achieved a complete response (CR) and 35% achieved a partial response (113).

### 4.3 Tislelizumab

Early results indicated that tislelizumab is generally well tolerated and has anti-tumor activity in patients with advanced solid tumors such as esophageal, gastric, hepatocellular and non-small cell lung cancer (141). A total of 674 patients with a minimum follow-up of 33 months were enrolled in a phase Ia/Ib study investigating tislelizumab. The primary endpoint of the study was overall survival, with secondary endpoints including objective response rate, progression-free survival, duration of response and safety. In this study, single agent tislelizumab demonstrated similar overall survival and significantly higher and longer lasting objective responses compared to sorafenib. However, sorafenib demonstrated better disease control rates and median progression-free survival. Tislelizumab demonstrated a favorable safety profile with no new safety concerns compared to sorafenib. Overall, these results suggest that tislelizumab may be a promising first-line treatment option for patients with unresectable HCC (115, 142). Tislelizumab is also currently being investigated in combination with sitravatinib as adjuvant therapy for HCC at high risk of recurrence after curative resection, and alone or in combination with levatinib as neoadjuvant treatment for resectable recurrent HCC (143, 144).

### 4.4 Toripalimab

Toripalimab is a selective, recombinant, humanized monoclonal antibody targeting PD-1 which is recently been approved for the treatment of metastatic or recurrent, locally advanced nasopharyngeal carcinoma in combination with cisplatin and gemcitabine (145). In terms of HCC, efficacy and safety of hepatic arterial infusion chemotherapy of oxaliplatin, 5-fluorouracil and leucovorin plus lenvatinib and toripalimab was evaluated. 36 patients were enrolled in this study, and the primary endpoint revealed 80.6% progression free 6 months survival rate. Eight patients were downstaged to resectable disease. Of these, one patient underwent liver transplantation and four underwent curative surgical resection. One patient achieved a pathologic complete response. In addition, all observed adverse events were reported to be manageable and no treatment-related deaths were reported (116).

### 4.5 Sintilimab

Sintilimab is a fully human IgG4 monoclonal antibody that targets PD-1and firstly has been recognized as treatment for classical Hodgkin’s lymphoma in the ORIENT-1 trial (146, 147). The ORIENT-32 study evaluated the safety, tolerability and efficacy of sintilimab in combination with the bevacizumab biosimilar IBI305 as first-line treatment in patients with HCC compared to sorafenib. The results showed that combinatorial treatment with sintilimab and IBI305 significantly increased overall survival and progression-free survival in the first-line setting for unresectable HBV-associated HCC with an acceptable safety profile (117). Efficacy and safety of Sintilimab in combination with levatinib was evaluated for local advanced HCC in a Phase 2 (118). In another Phase 2 trial, combinatorial treatment of donafenib and sintilimab was evaluated in patients with advanced HCC (148).

### 4.6 Camrelizumab

Camrelizumab is a humanized anti-PD-1 monoclonal antibody that differs from nivolumab and pembrolizumab in terms of its target epitopes. Anticancer activity and safety of camrelizumab was evaluated in pretreated patients with advanced HCC (119). Among a total of 217 patients who received camrelizumab, 32 (14.7%) had an objective response and the overall survival probability at 6 months was reported to be 74.4%. In conclusion, camrelizumab demonstrated efficacy against previously untreated advanced HCC with manageable side effects, suggesting that it may be a promising novel therapeutic option for these patients (119).

### 4.7 Spartalizumab

Spartalizumab is a humanized IgG4 monoclonal antibody that binds PD-1 (120). A Phase ½ study evaluated the safety and efficacy of spartalizumab in combination with the selective FGFR4 inhibitor FGF401 in patients with FGFR4/KLB expressing tumors, including HCC. The results showed that FGF401 alone or in combination with spartalizumab was safe in patients with FGFR4/KLB-positive tumors (120).

### 4.8 Cemiplimab

Cemiplimab is a recombinant human IgG4 monoclonal antibody targeting the PD-1 receptor with potent anticancer activity and a safety profile comparable to other anti-PD-1 therapies (149). A Phase 1 study evaluated the safety, tolerability and antitumor activity of cemiplimab in patients with unresectable HCC who had progressed, were intolerant or declined first-line systemic therapy. Of the 26 patients evaluated, 5 (19.2%) showed a partial response, 14 (53.8%) were stable and 6 (23.1%) had progressive disease, while 1 patient was not evaluable. Of note, only 5 patients (19.2%) completed the planned 48 weeks of treatment, while the remaining patients discontinued treatment prematurely, mainly due to disease progression (121). In a Phase 2 study, patients with resectable HCC received neoadjuvant cemiplimab intravenously every 3 weeks, followed by surgical resection. Twenty-one patients were enrolled in the trial, and all received neoadjuvant cemiplimab. Successful tumor resection was achieved in 20 patients. Of these, 4 patients (20%) had significant tumor necrosis. 3 (15%) of the patients who underwent resection had a partial response, while the remaining patients had stable disease. Throughout the neoadjuvant treatment period, 95% of patients experienced treatment-emergent adverse events of various grades (122).

### 4.9 Atezolizumab

Atezolizumab, the first FDA-approved anti-PD-L1 antibody, is a fully human IgG1 monoclonal antibody used in combination with the VEGF inhibitor bevacizumab in HCC. In a Phase 1b study, patients with unresectable HCC who had not received prior systemic therapy who received a combination of atezolizumab and bevacizumab had longer progression-free survival compared to those who received atezolizumab alone (123). In IMbrave150 trial, efficacy and safety of atezolizumab-bevacizumab combination was compared with sorafenib in participants with locally advanced or metastatic HCC who have received no prior systemic treatment (124). The results revealed that atezolizumab combined with bevacizumab favored overall and progression-free survival outcomes compared to sorafenib in unresectable HCC.

### 4.10 Durvalumab

Similar to atezolizumab, durvalumab is a human IgG1 monoclonal antibody that targets PD-L1 and has received accelerated approval for the treatment of locally advanced or metastatic urothelial carcinoma (150). The safety and efficacy of durvalumab in combination with radioembolization with yttrium-90 microspheres were evaluated in locally advanced and unresectable HCC. Transarterial radioembolization (TARE) with yttrium-90 microspheres was administered in combination with 1500 mg intravenous (IV) durvalumab every 4 weeks. Of the 24 patients enrolled, seven (29.2%) had a complete response and 13 (54.2%) had a partial response, while none of the participants experienced any treatment-related serious adverse events. These results suggest that this treatment modality has shown promising efficacy and safety in patients with locally advanced unresectable HCC (125).

A Phase 2 trial evaluated durvalumab and the anti-CTLA-4 monoclonal antibody tremelimumab or durvalumab in combination with tremelimumab or bevacizumab for the treatment of patients with unresectable HCC where the results indicated that combinatorial durvalumab and bevacizumab showed promising clinical safety and efficacy (126).

In a Phase 3 study, HIMALAYA trial, durvalumab and tremelimumab combination therapy and durvalumab monotherapy versus sorafenib in the treatment of patients with no prior systemic therapy for unresectable HCC is evaluated. HIMALAYA is unique in that it is the first large Phase 3 trial to enroll a diverse and representative population of patients with unresectable HCC and to include extensive long-term follow-up to evaluate the efficacy of both monotherapy and combination immunotherapy approaches. Outcomes of this study revealed that durvalumab was noninferior to sorafenib with favorable safety; and the combinatorial administration of tremelimumab plus durvalumab may be considered as a first-line standard of care systemic therapy for unresectable HCC (127). Several clinical trials are currently being conducted with durvalumab, either as a monotherapy or in combination, for the treatment of HCC (151, 152).

### 4.11 Avelumab

Avelumab is a fully human IgG1 monoclonal antibody that is directed against PD-L1 (153). In a Phase 2 study, avelumab was evaluated in patients with advanced HCC following treatment with sorafenib. A total of 30 patients were enrolled and received 10 mg/kg avelumab every 2 weeks until disease progression or unacceptable toxicity. The primary endpoint of the study was objective response rate, and secondary endpoints included time to progression, overall survival, disease control rate and safety. However, no complete responses were observed, while three patients (10.0%) had partial responses. In conclusion, avelumab was well tolerated and showed moderate efficacy in advanced HCC previously treated with sorafenib (128).

A Phase 1b study, VEGF Liver 100, evaluated the safety and efficacy of avelumab plus TKI axitinib in treatment-naive patients with advanced HCC. Of the 22 patients enrolled, 16 patients (72.7%) experienced grade 3 treatment-emergent adverse events and 10 patients (45.5%) experienced immune-related adverse events. There were no treatment-related deaths. The objective response rate was 13.6%. These results suggest that avelumab plus axitinib has anti-tumor activity with a manageable toxicity profile in advanced HCC, which was also consistent with the established safety profiles of avelumab and axitinib when administered alone (129). The activity of TACE and stereotactic body radiotherapy followed by avelumab was evaluated in a Phase 2 study (START-FIT) in advanced unresectable HCC. A total of 33 patients were enrolled in this study; 11 (33%) experienced treatment-emergent adverse events and five (15%) patients experienced grade 3 or higher immune-related adverse events (130).

### 4.12 Ipilimumab

Ipilimumab is a fully human monoclonal IgG1 antibody targeting CTLA-4 (154). Based on cohort 4 of CheckMate 040 trial, ipilimumab in combination with nivolumab has been approved by the FDA for the treatment of HCC in patients who have received prior treatment with sorafenib (131). An ongoing Phase 2 trial is evaluating the efficacy of ipilimumab in combination with nivolumab in patients with advanced HCC who have progressed after first-line treatment with atezolizumab and bevacizumab (155). In another ongoing Phase 2 study, it is aimed to investigate efficacy of ipilimumab/nivolumab and TACE in patients with HCC who are not eligible for curative intent treatment (156).

### 4.13 Tremelimumab

Tremelimumab, a fully human monoclonal antibody targeting CTLA-4, was initially evaluated as a checkpoint inhibitor in patients with HCC and chronic HCV infection. The study included 21 patients, of which 3 patients discontinued the study. Among the 17 patients evaluated for tumor response, the overall response rate was 17.6%. Importantly, the treatment was generally well tolerated with only a small number of patients experiencing significant adverse events (132). In a Phase 1/2 study, tremelimumab with chemoembolization or ablation was evaluated for HCC treatment. a total of 61 patients were enrolled in this study, and the results indicated that tremelimumab promotes activation of T cell responses in HCC and in combination with tumor ablation, it can be regarded as a potential novel treatment for patients with advanced HCC (133, 134). Another Phase 1/2 study evaluated the safety and efficacy of tremelimumab in combination with durvalumab; of the 40 patients enrolled, all patients had a partial response and six (15%) had an overall response. No unexpected safety signals with durvalumab and tremelimumab were observed (135).

### 4.14 Cobolimab

Increased expression of TIM-3 on monocytes in individuals with chronic HBV suggests that patients with HCC have increased expression of TIM-3 on peripheral blood monocytes compared to controls. Furthermore, there appears to be a negative correlation between TIM-3 expression and patient survival, highlighting the potential importance of TIM-3 in HCC prognosis (157). A Phase 2 study is currently evaluating the anti-TIM-3 antibody cobolimab in combination with dostarlimab in advanced HCC, which is expected to be completed in 2025. The study is designed to enroll 42 patients diagnosed with histologically confirmed HCC at BCLC stage B or C. Participants will receive cobolimab 300 mg and dostarlimab 500 mg on the first day of each 21-day cycle. Interim results indicate that the combined regimen of cobolimab and dostarlimab has an acceptable safety profile with encouraging clinical activity as a first-line treatment in patients with advanced HCC (136).

## 5 Considerations of immune checkpoint inhibition in hepatocellular carcinoma treatment

The liver is characterized by a distinct immunological milieu, with the presence of immune cells predisposed to promote tolerance and immune suppression. Given the constant exposure of the liver to foreign antigens and bacterial by-products in the portal blood, is advantageous for the maintenance of normal biological function. Unfortunately, this tolerogenic state within the liver creates a conducive environment for the initiation and progression of both primary and metastatic liver tumors. The suppressive nature of intrahepatic immune cells represents a significant barrier to the development of effective anti-tumor immunotherapy strategies. Thus, deeper understanding of liver immune cell biology is essential to pave the way for innovative immunotherapeutic approaches tailored to combat liver tumors (158).

Including HCC, cancers often display a heterogeneous composition of immune cells within the TME, exhibiting variations in type, density, and spatial distribution. The established immunoheterogeneity pattern, which is particularly relevant to the efficacy of ICIs, categorizes tumors into three distinct profiles: hot, excluded, and cold. Hot tumors have an abundance of T cells actively engaged in anticancer activities, making them more likely to respond favorably to ICIs. Conversely, cold tumors lack T cells, indicating a reduced likelihood of a robust response to immunotherapy. In between these extremes, immune-excluded tumors exhibit an intermediate responsiveness to ICIs; here, T cells predominantly accumulate at the tumor periphery and are unable to effectively infiltrate the core. This simplified, yet powerful conceptualization serves as a predictive framework for the therapeutic outcomes of ICIs in various malignancies (159). To overcome resistance to HCC immunotherapy, it may be advantageous to identify targets capable of transforming the TME from immunologically cold to hot in order to enhance their responsiveness to immunotherapy (31). For this purpose, combination therapies may enhance the efficacy of ICI in HCC. An example of this approach may be the combinatorial administration of anti-VEGF antibodies with ICI. Since HCC is a highly vascularized tumor, targeting angiogenesis has emerged as a promising avenue for therapeutic intervention. In addition, VEGF exerts inhibitory effects on the immune response by affecting cytotoxic T cells, DCs, Tregs and MDSCs (160–162). In this context, the combination of atezolizumab and the anti-VEGF antibody bevacizumab is a pioneering systemic therapy which does not only inhibit angiogenesis, but also demonstrates an overall survival benefit that exceeds that of conventional sorafenib, marking a significant advancement in the therapeutic landscape for patients with unresectable HCC (124, 163).

Because liver tumors typically harbor multiple immunosuppressive factors, isolated blockade of a single factor appears insufficient to achieve substantial improvements. Therefore, simultaneous inhibition of non-redundant immunosuppressive pathways is expected to provide superior efficacy compared to singular blockade of one immune checkpoint. Consistent with this, inhibition of the PD-1 and CTLA-4 pathways by administering nivolumab in combination with ipilimumab demonstrated a manageable safety profile and achieved an objective response rate of 32% in patients with advanced HCC previously treated with sorafenib (164). Another study in HCC patients who progressed on prior single-agent ICI therapy showed that dual ICI therapy with ipilimumab in combination with either nivolumab or pembrolizumab resulted in durable anti-tumor responses and encouraging survival outcomes (165).

Currently, TACE, a versatile approach that can be tailored to the specific stage of diagnosis and incorporates techniques such as angiography and computed tomography (CT), is widely accepted as the primary and effective treatment for HCC patients with intermediate stage HCC (166, 167). This method is known for its interdisciplinarity, allowing the combination with various treatments such as radiotherapy, percutaneous ethanol injection and RFA. The release of tumor-associated antigens during all types of locoregional therapy, including TACE, can stimulate immune responses and ideally lead to a synergistic effect of both therapies. Similarly, thermal ablation has been reported to promote inflammation and increase tumor antigens to induce a cancer-immunity cycle and act synergistically with ICI. Both preclinical and clinical research has provided compelling evidence supporting the combination of ICI with thermal ablation as a means to reverse T-cell depletion, however, despite this promising potential, the clinical feasibility of activating immune responses through a combination of ICI monotherapy and thermal ablation appears to be limited as this approach is not widely used in clinical practice (168). In summary, eliminating HCC by ablation may activate the immune system, which can potentially recognize and kill remaining cancer, while ICIs may also enhance this effect (167).

Another area to be explored is biomarkers to predict the ICI treatment efficacy in HCC. In IMbrave150 trial in which the patients were administered atezolizumab plus bevacizumab, pre-existing immunity was characterized by intratumoral CD8+ T cell density, high expression of CD274 encoding PD-L1, and T effector signature were favorably associated with the outcome; whereas a high Treg to effector T cell ratio and high expression of oncofetal genes (GPC3 and AFP) were associated with reduced benefit from the combination therapy (169). In another study aiming to unravel molecular markers that correlate with ICI response in hot tumors, the authors demonstrated that none of the cold or excluded tumors responded to ICI therapy. Interestingly, half of the hot tumors were also reported to be unresponsive. Further analysis revealed an enrichment of terminally exhausted T cells in non-responders, and the presence of intratumoral DC-CD4+ T helper cell niches was reported to promote the efficacy of ICI therapy (170).

Besides TME, the important role of the gut microbiota in regulating systemic immunity and influencing responses to immunotherapy and the immune effect of chemotherapy is widely accepted. Similarly, in ICI therapy, the diversity of the host microbial flora has been shown to influence clinical outcomes in HCC. In addition, the dynamic changes in gut microbiome characteristics hold the potential for early prediction of immunotherapy outcomes. Understanding the impact of the microbiota on the response to ICI, coupled with evidence from preclinical studies demonstrating HCC prevention through antibiotic-induced modulation of the gut microbiota may form the basis for considering clinical trials exploring the combination of immunotherapies with antibiotics or probiotics (171, 172).

Finally, ICIs may be recognized as non-self by the host immune system and may induce the generation of anti-drug antibodies (ADAs). While ADA formation upon treatment has been studied extensively, data on their clinical significance remains limited, yet it is known that ADAs can reduce drug availability which may result in decreased clinical efficacy (173). In the IMbrave150 study, ADA positivity resulted in decreased treatment efficacy compared to those who did not develop ADAs, most likely due to an increased rate of drug clearance (20). In another study, ADA-positive patients who received atezolizumab plus bevacuzimab for 3 weeks had worse progression-free survival and overall survival compared to placebo (174). In this study, high ADA levels were reported to be positively correlated with impaired CD8+ T cell proliferation and decreased IFN-γ and TNF-α production by CD8+ T cells. All these findings suggest that monitoring ADA formation during treatment regimens that include ICI, not only in HCC but in all malignancies, may improve the safety and efficacy of therapy, in addition to aiding clinicians in determining the ideal combinatorial treatment regimen for their patients (174).

## 6 Conclusion

In recent years, immunotherapy has brought about a significant and lasting change in the field of systemic therapy for patients with advanced HCC, as the results of numerous phase 2 and 3 trials have shown promising results with the administration of PD-1, PD-L1 and CTLA-4 ICIs. Nevertheless, phase 3 trials evaluating ICI monotherapies versus TKIs as first- or second-line treatment have yielded conflicting results, though they have encouraged further investigation into this therapeutic modality. Moreover, clinical trials of combinatorial administration of ICIs with other targeted therapies in addition to TKIs for the second-line treatment of advanced HCC are ongoing and showing promising results (111, 175). In summary, ICIs hold great promise for becoming the standard of care for HCC treatment in the future. On the other hand, a tumor’s response to ICI is strongly influenced by its immune cell composition, so therapeutic interventions aimed at converting “cold” tumors into “hot” tumors may enhance the efficacy of ICI-based therapies. In addition, further studies focused on elucidating biomarkers predictive of ICI treatment response may help to select the optimal patient population that may benefit from ICI. Last but not least, routine monitoring during ICI administration may help clinicians to select the ideal drug combination while increasing the efficacy of the treatment.

## Author contributions

ZA: Conceptualization, Data curation, Investigation, Writing – original draft. BA: Conceptualization, Data curation, Investigation, Visualization, Writing – original draft. FA: Visualization, Writing – original draft. GYD: Writing – review & editing.
